# Soil C, N, and P distribution as affected by plant communities in the Yellow River Delta, China

**DOI:** 10.1371/journal.pone.0226887

**Published:** 2019-12-20

**Authors:** Shuying Jiao, Junran Li, Yongqiang Li, Jiwen Jia, Ziyun Xu

**Affiliations:** 1 College of Resources and Environment, National Engineering Laboratory for Efficient Utilization of Soil and Fertilizer Resources, Shandong Agricultural University, Tai’an, Shandong, China; 2 Department of Geosciences, The University of Tulsa, Tulsa, OK, United States of America; Shandong University, CHINA

## Abstract

Soil carbon (C), nitrogen (N) and phosphorus (P) are important soil properties linked to nutrient limitation and plant productivity in terrestrial ecosystems. Up to 90% of the Yellow River Delta (YRD), China has been affected by soil salination due to groundwater overdraft, improper irrigation, land use and land cover change. The objective of this study is to evaluate the impact of different plant communities on soil quality in a saline-alkaline system of the YRD. We investigated the vertical distribution and seasonal variation of soil C, N, and P, and C:N ratio by choosing four dominant plant communities, namely, alfalfa grassland (AG), Chinese tamarisk (CT), locust forest (LF) and cotton field (CF). The results showed that the concentrations of soil organic carbon (SOC) and total nitrogen (TN) in CT and LF were always higher than that in AG and CF, especially in the topsoil layer (*p*<0.05), then gradually decreased with soil depth increasing (*p*<0.05). The C:N ratio was generally lower, and the average C:N ratio was higher in LF (11.55±1.99) and CT (11.03±0.47) than in CF (10.05±1.25) and AG (9.11±1.11) (*p*<0.05). The available phosphorus (AP) was highest in CT in Spring, while it was highest in CF in Summer and Autumn. It is worth noting that the soil AP concentrations were always low, particularly in AG (< 6.29 mg kg^-1^) and LF (< 4.67 mg kg^-1^), probably linked to P poorly mobile in the saline-alkaline region. In this study, soil nutrients in natural plant communities are superior to farmland, and are significantly affected by the types of plant community; therefore, we suggest that protection of natural vegetation and development of optimal vegetation are critical to restoring land degradation in the YRD.

## Introduction

Soil nutrient concentration is an important index for estimating soil fertility and determining ecological function. Soil carbon (C), nitrogen (N) and phosphorus (P) are important indicators linked to nutrient limitation and plant productivity in terrestrial ecosystems. The soil C, N and P stoichiometry can improve the understanding of the ecological processes of nutrient circulation and ecosystem responses to climate change and disturbance [1,2], which could regulate vegetation patterns, atmospheric C and N composition, optimal plant growth and ecosystem functioning [3–5].

C, N and P are important components of agricultural soil. Understanding their characteristics can help us know about how ecosystems would respond to natural and anthropogenic disturbances under different vegetation conversion. For instance, soil organic carbon (SOC) is the key functional component which directly affects soil fertility, the production capacity of ecological systems, and plays an important role in the ecosystem responses to the environment [6–8]; its dynamics changes have a significant impact on the global carbon cycle. Soil N is the main N source for plants and acts as an important growth-limiting nutrient for plants [9, 10]; its concentration determines N pools in terrestrial ecosystems [11]. Soil P is low and poorly mobile in most regions and is also a critical growth-limiting element for plants [11, 12]. The available P can be directly absorbed and utilized by plants; it is the main form of nutrient phosphorus absorbed by vegetation and an important index for evaluating soil phosphorus supply capacity. The availability of soil P involves complex biotic and abiotic processes [3, 13], and P fertilization can lead to eutrophication of water by P runoff and leaching [9, 14].

The balances of soil C, N, and P have been shown to be affected by environmental factors that promote global change, e.g., the increase in CO_2_ concentration, climate change, altered precipitation, changed plant communities [3, 15–17]. The soil C, N and P ratio directly reflects soil fertility, determines whether nutrients are immobilized or mineralized to be available for uptake, and indirectly determines plant nutritional status and productivity [1, 18]. The availability and limitation of N and P can feed back to soil C dynamics [19, 20]. Therefore, the ecological characteristics of soil C, N, and P are always one of the frontier hotspots in biogeochemical cycling and ecology research; it can lead to an improved understanding of soil-plant interactions and C, N and P cycles, and plays important roles in ecosystem dynamics and functioning. Many studies have reported that the concentration and distribution of soil nutrients can vary among soil properties and diverse plant communities for terrestrial and coastal grasslands [21, 22]. Some studies have found that soil C and N concentrations in wetlands varied by flow-sediment regulation [23] and multiple drying and wetting cycles improve SOC decomposition [24]. However, few studies have examined the effect of salination-affected plant communities on the distribution and seasonal dynamics of soil C, N and P.

The salination-affected area of the Yellow River Delta (YRD), China has experienced a dramatic disturbance due to land uses and anthropogenic activities, and has formed great variations in the vegetation types. Up to 90% of the YRD has been affected by soil salination due to groundwater overdraft and improper irrigation as well as land uses and vegetation cover changes; soil degradation was serious due to the conversation of vegetation types by frequent land use changes. Therefore, understanding the effects of different plant communities on the ecological characteristics of soil C, N and P in salination-affected areas of the YRD is very important for evaluating the ecological consequences due to land use changes and anthropogenic activities. In this study, we focused on the distribution variation and seasonal dynamics of soil C, N, and P, and C:N in the four dominant plant communities in the scale of the YRD. The objectives of our study were: (1) to examine the profile distribution and seasonal dynamics of soil C, N and P as affected by different plant communities; and (2) to characterize how soil C:N varies among soil profiles and plant communities in a salination-affected area. Based on this research, we will explore which plant communities should be optimally developed and how to manage land uses to restore land degradation in the salination-affected areas of the YRD, China.

## Materials and methods

### Ethics statement

Both of field activities (alfalfa grassland: AG and cotton field: CF) were conducted with the permission of the farmers who owned the land we sampled, both of natural forests (Chinese tamarisk: CT and locust forest: LF) did not require specific permission, and we confirmed that the field studies did not involve any endangered or protected species.

### Study area

**Fig 1:**
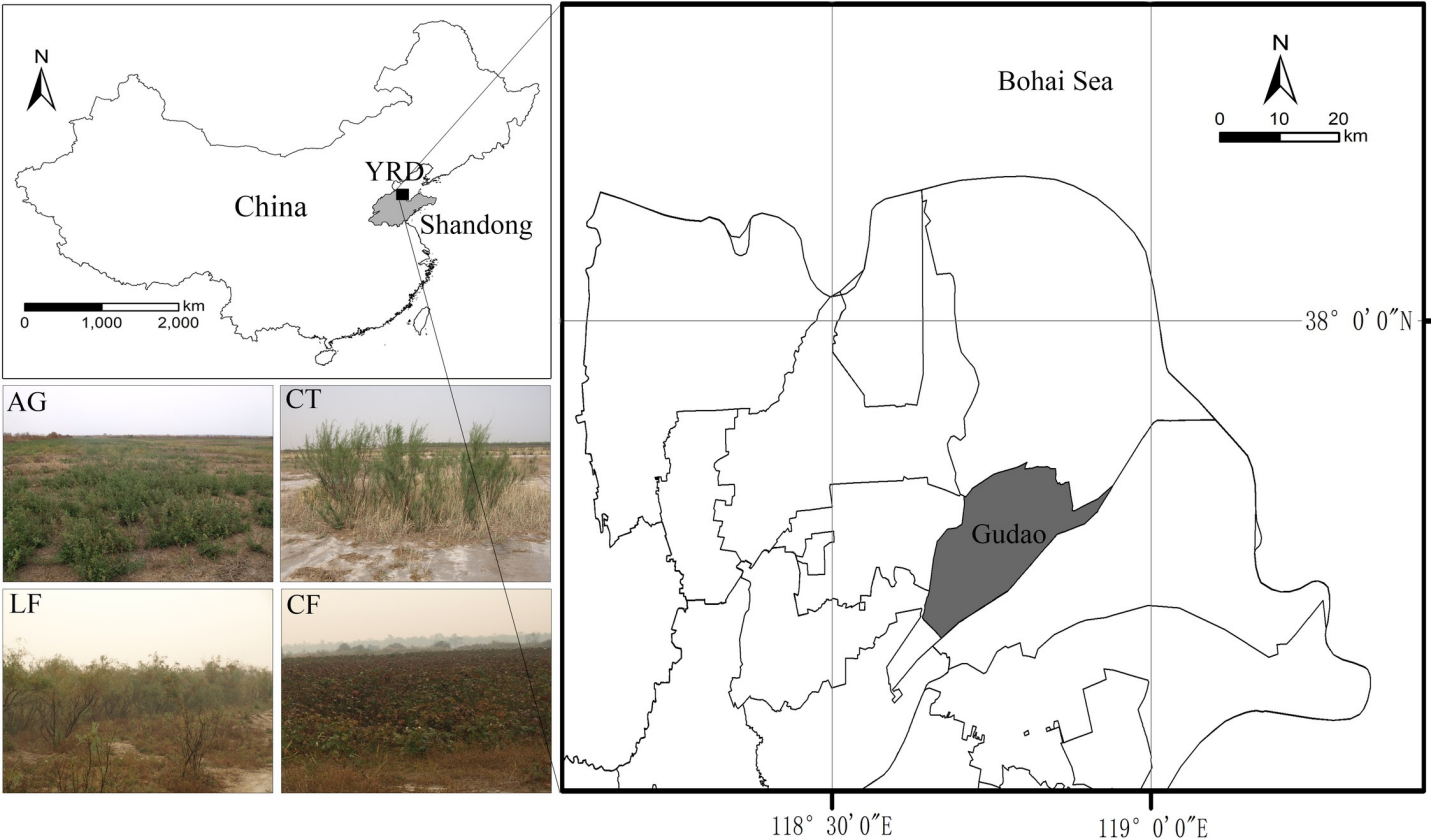
The study location and plant communities of the northeast estuarine area in the YRD, China.

The study site is located in the Gudao town of the estuary region, YRD, Shandong Province, China (E118°42′24.8′′; N 37°46′57.4′′) (Fig 1), which experiences a semi-humid, warm-temperate, continental monsoon climate. The annual mean precipitation is 692 mm, and the evaporation is approximately 1900–2000 mm, with 63.9% of the total precipitation occurring during July and August. The mean air temperature is 12.3°C. Soil is the parent material of alluvial loess, and mainly includes salinized fluvo-aquic soil (Salic Fluvisols) and coastal saline soil (Gleyic Solonchaks) (FAO,1999). The predominant plant communities in the study area include two kinds of forest: *Tamarix chinensis* Lour. and *Robinia pseudoacacia* L., artificial grassland: *Medicago sativa* L. and cotton field: *Gossypium hirsutum* L.. *T*. *chinensis* forest is the natural forestland, with light grazing utilization, which belongs to a natural secondary forest; *Phragmites australis* (Cav.) Trin. ex Steud. and *Suaeda heteroptera* Kitag. are the main understory plants, with a coverage of approximately 18%. *R*. *pseudoacacia* was planted in the 1960s, and its canopy density is 77%; there are abundant understory herbs, such as *Setaria viridis* (L.) Beauv., *Carex lanceolata* Boott., *P*. *australis*, *Imperata cylindrica* (L.) Beauv., *S*. *heteroptera* and *Digitaria sanguinalis* (L.) Scop.. The artificial grassland is purple alfalfa (*M*. *sativa*), reclaimed more than 10 years ago, with three cuts every year. The cotton field is artificially cultivated, with 85% coverage. It has been planted for more than 10 years, with fertilizer applied every year.

### Experimental design

We selected four representative plant communities within the same physiographical units in the study region, including alfalfa grassland (AG), Chinese tamarisk (CT), locust forest (LF) and cotton field (CF) (Fig 1 left). Four 20 m × 20 m plots were established at each plant community respectively and were considered as true replicates, the distance between each plot exceeded the spatial dependence (<13.5 m) of most soil physicochemical and microbiological properties variables [25, 26]. Soil samples were collected by using a soil core sampler (3.8 cm-diameter) in Spring (29 March), Summer (8 July) and Autumn (22 October) of 2014. Five points were sampled according to an “S” curve in each plot at six depths (0–10 cm, 10–20 cm, 20–30 cm, 30–40 cm, 40–50 cm, 50–60 cm) and mixed to create a representative soil sample. All of the mixed samples were air-dried and crushed to pass through a 0.25-mm sieve. Soil samples were analyzed for soil organic carbon (SOC), total nitrogen (TN), available phosphorus (AP), and available potassium (AK), pH and electrical conductivity (EC).

### Laboratory analysis

SOC and TN were measured by the K_2_C_r2_O_7_-H_2_SO_4_ oxidation method [27, 28] and semi-micro Kjeldahl digestion procedure [29] respectively. AP was extracted with 0.5 M NaHCO_3_ (pH 8.5), AK was determined by the ammonium acetate extract-flame photometric method. The pH was determined with a pH meter (Sartorius PB-10) in a 1:2.5 (w/w) soil-to-water suspension, EC was measured by a conductivity meter (DDS-307) with a water-soil ratio of 5:1 [30]. Total C concentrations were equal to soil organic carbon (Corg) [31–33], since the measured inorganic carbon concentrations of the samples were almost nil. The C:N ratios were calculated as atomic mole ratios using SOC:TN data [23, 34].

### Data analysis

The seasonal mean values used in this study were calculated from the monthly mean values, which were first averaged from all soil layer measurements in the same month among the four plant communities. Differences in C, N, P and C:N ratio among plant communities and soil layers were examined using one-way analysis-of-variance (ANOVA), with a Duncan multiple-range procedure test at *p*≤0.05, using SPSS 16.0 Software (SPSS Inc., Chicago, IL). Correlations between soil C, N and P were examined for each plant community using Tukey’s honest significant differences (HSD) test, with a 95% confidence interval. All figures and tables were constructed using Origin 10.0 and Excel 2013, respectively.

## Results

### Soil C, N, and P concentrations and seasonal dynamics

**Fig 2:**
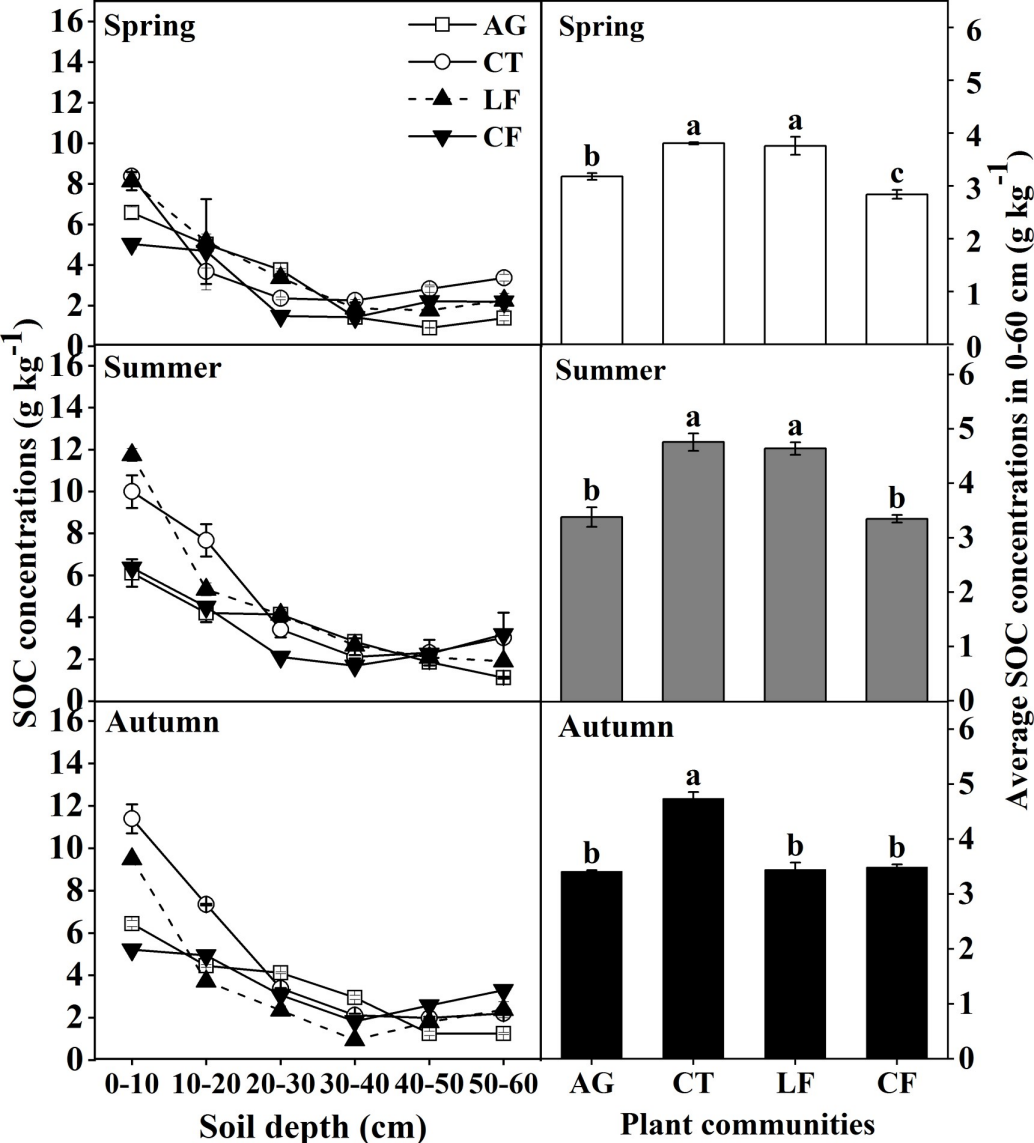
The distribution of SOC concentration at varying soil depths in the 0–60 cm layer. Bars indicate a significant difference (n = 4, *p*<0.05) at the same soil depths between different plant communities, as determined by ANOVA; AG: alfalfa grassland, CT: Chinese tamarisk, LF: locust forest and CF: cotton field.

SOC concentrations varied significantly between plant communities in each soil layer during the same season (*p*<0.05) (Fig 2). The highest SOC concentrations in the soil surface of 0–10 cm were in CT (8.37 g kg^-1^ in Spring and 11.39 g kg^-1^ in Autumn) and in LF (11.74 g kg^-1^ in Summer). SOC concentration was higher in CT than in other plant communities in each 0–60 cm layer and decreased slowly with soil depth; it decreased gradually with soil depth but increased in AG and LF; a “V” trend was observed in CF, with the lowest SOC concentration in the 30–40 cm soil layer. The average SOC concentrations in CT and LF in the 0–60 cm layer were higher than those in AG and CF in Spring and Summer. However, it was highest in CT in Autumn (*p*<0.05).

**Fig 3:**
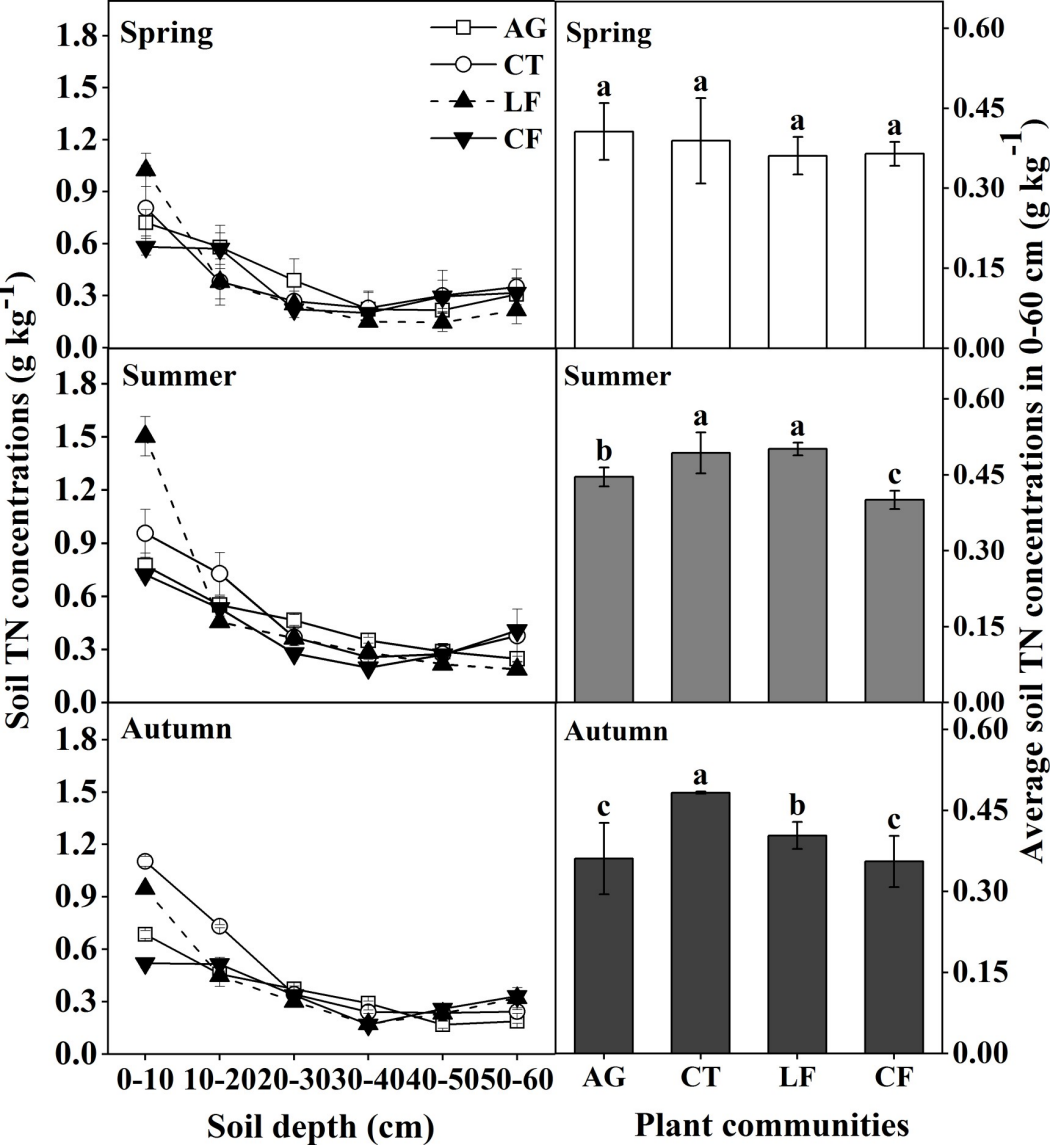
The distribution of soil TN concentration at varying soil depths in the 0–60 cm layer. Bars indicate a significant difference (n = 4, *p*<0.05) at the same soil depth between different plant communities, as determined by ANOVA; AG: alfalfa grassland, CT: Chinese tamarisk, LF: locust forest and CF: cotton field.

Soil TN concentrations in the 0–10 cm layer were highest in LF and were 1.02 g kg^-1^ in Spring and 1.50 g kg^-1^ in Summer, respectively. It was highest (1.10 g kg^-1^) in CT in Autumn, while it was less than 1 g kg^-1^ in other plant communities and different seasons (Fig 3). The average soil TN concentration in the 0–60 cm layer was also higher in LF in Summer and was higher in CT in Autumn.

**Fig 4:**
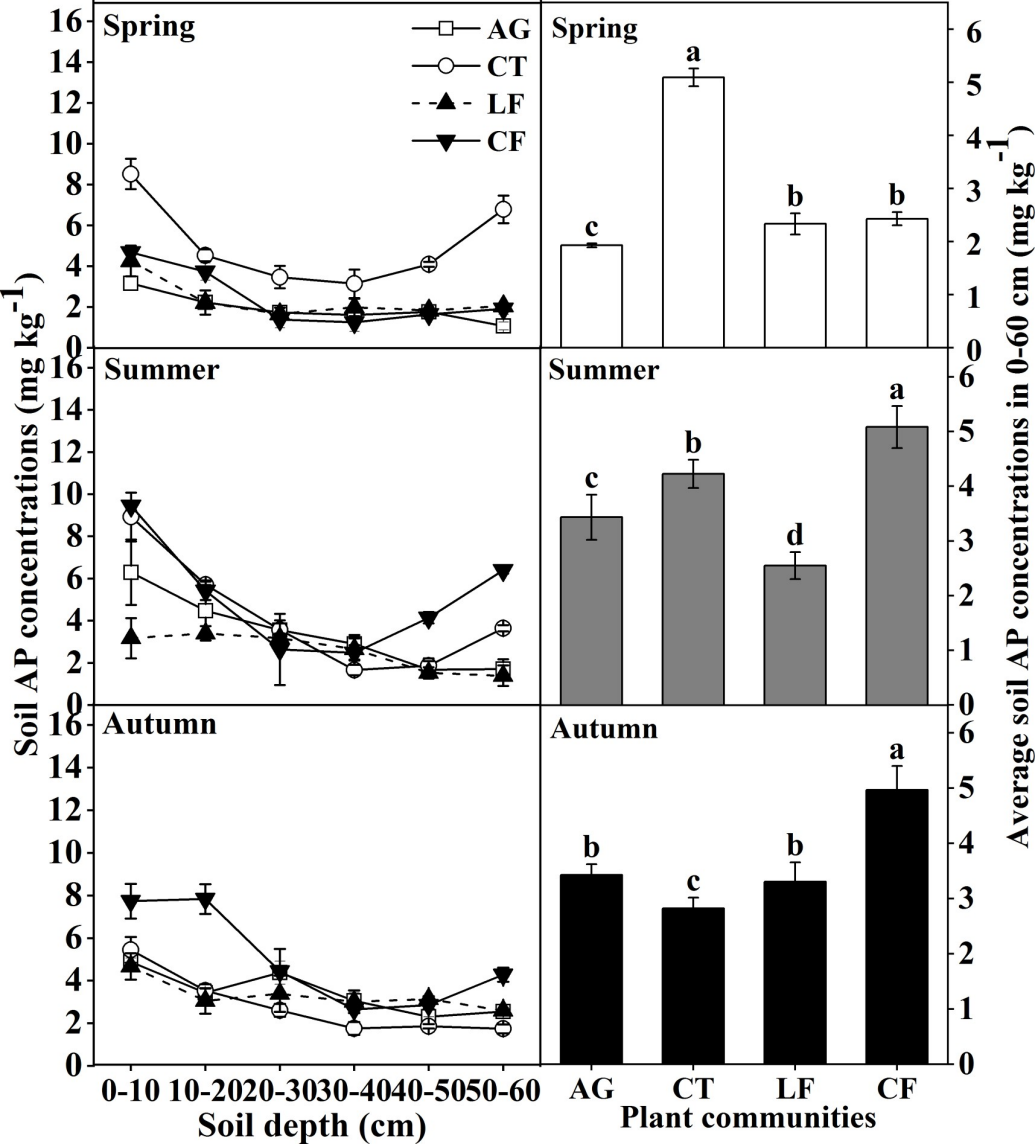
The distribution of soil AP concentration at varying soil depths in the 0–60 cm layer. Bars indicate a significant difference (n = 4, *p*<0.05) at the same soil depth between different plant communities, as determined by ANOVA; AG: alfalfa grassland, CT: Chinese tamarisk, LF: locust forest and CF: cotton field.

Soil AP concentration in CT was the highest in the 0–60 cm layer in Spring, but it increased in CF in Summer and Autumn; it changed slowly in each 0–60 cm layer in LF, among different seasons (Fig 4).

**Fig 5:**
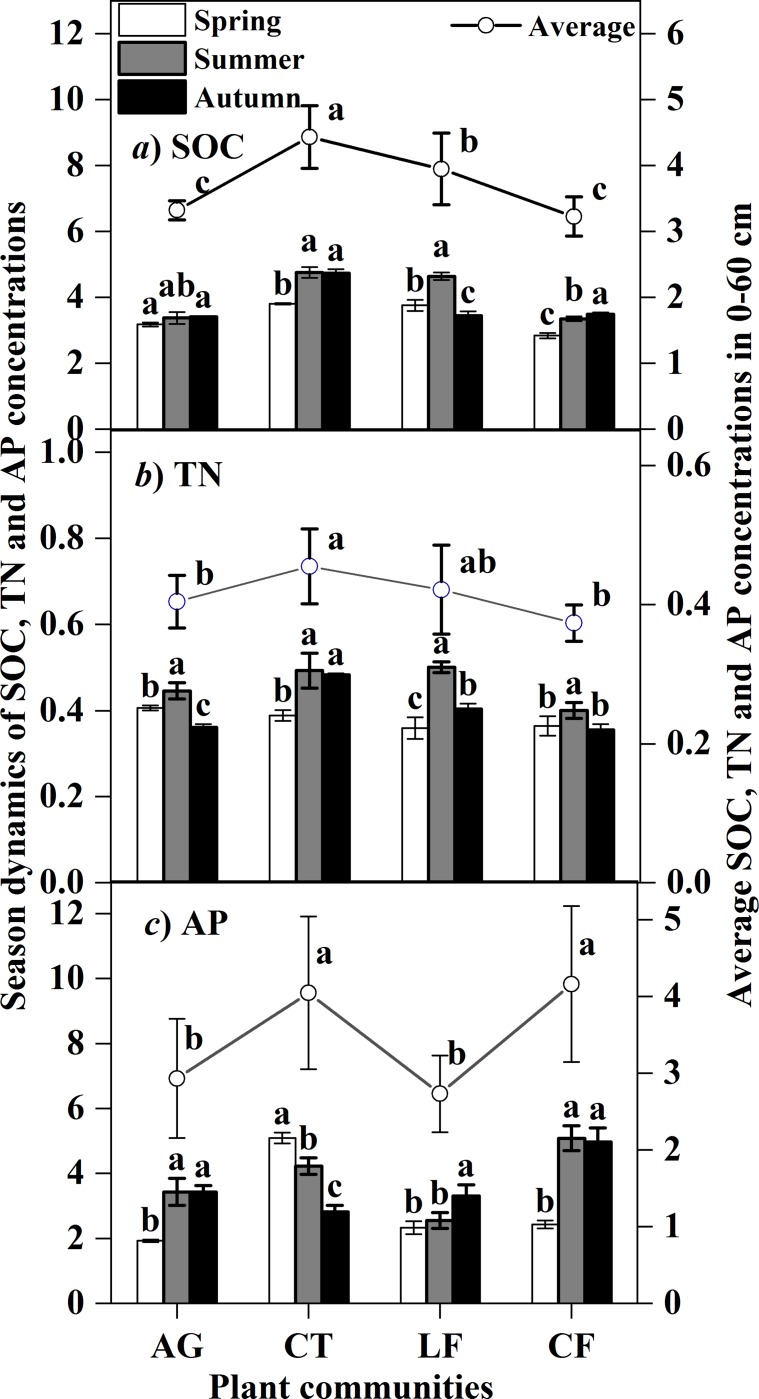
Seasonal dynamics and average concentrations of SOC, TN and AP. Bars indicate a significant difference (n = 4, *p*<0.05) between different seasons and between plant communities, as determined by ANOVA; AG: alfalfa grassland, CT: Chinese tamarisk, LF: locust forest and CF: cotton field.

SOC concentrations varied with the seasons, except for in AG (Fig 5*A)* SOC). Concentrations were significantly higher in Summer and Autumn in CT and CF and were lower in LF in Autumn. The average SOC of three seasons was highest in CT (4.43 g kg^-1^) in the 0–60 cm layer. The seasonal dynamics of soil TN concentration exhibited almost the same trend as SOC, but the average TN concentration between plant communities was not significantly different (*p*>0.05) (Fig 5*B)* TN). Soil AP concentrations were increased significantly in AG and CF in Summer and in LF in Autumn (Fig 5*C)* AP), but it decreased with seasonal changes in CT. The average soil AP concentration in plant communities was higher in CT and CF than in AG and LF.

### Ratio of soil carbon to nitrogen (C:N)

**Fig 6:**
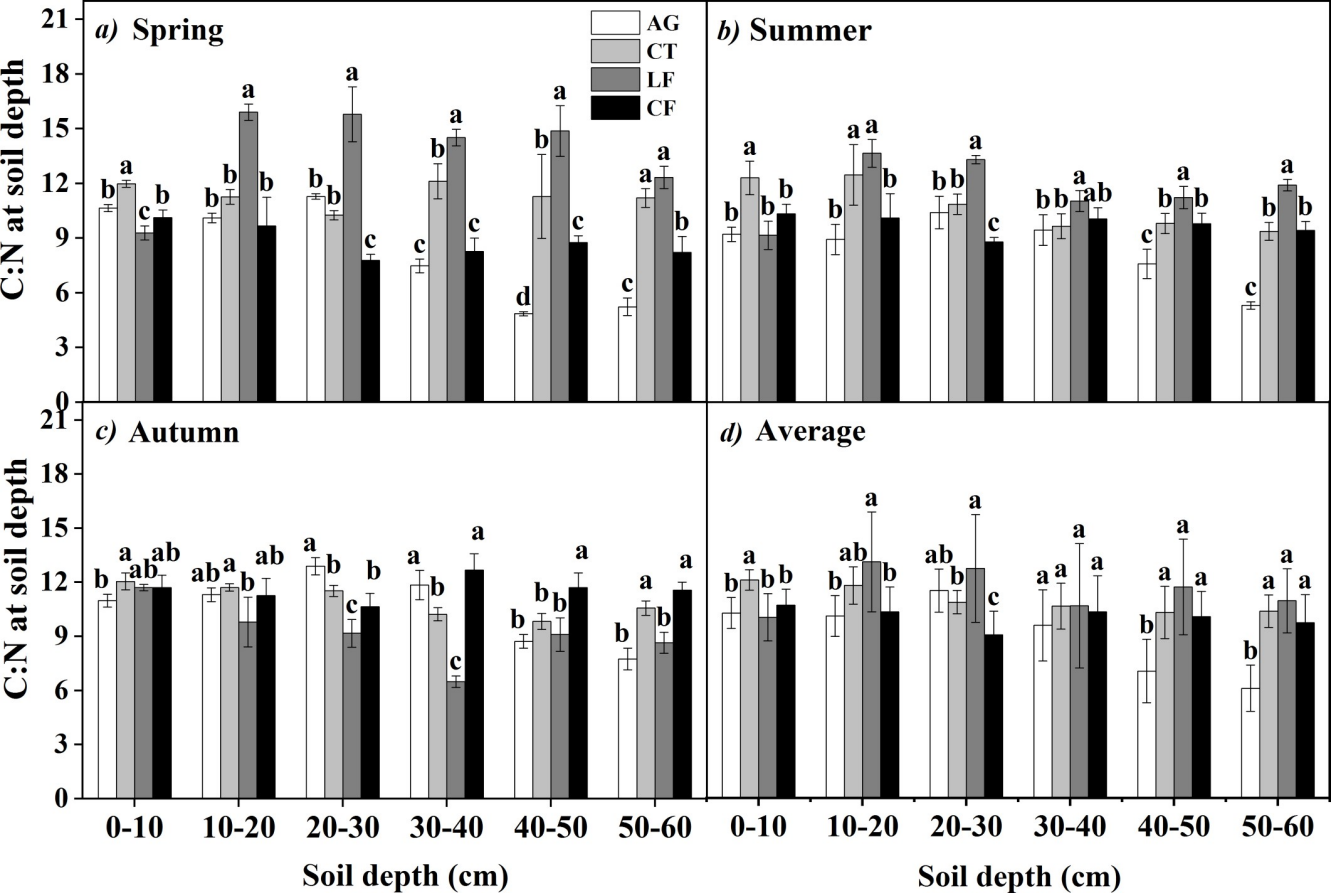
The ratio of soil carbon to nitrogen (C:N) among plant communities. Bars indicate a significant difference (n = 4, *p*<0.05) between plant communities at the same soil depth, as determined by ANOVA; AG: alfalfa grassland, CT: Chinese tamarisk, LF: locust forest and CF: cotton field.

The soil C:N ratio varied significantly between different plant communities at the same soil depth in each season (Fig 6). There was a different C:N ratio trend with increasing soil depth. The C:N ratio in AG and CF showed an increasing trend across seasons, particularly in the 20–30 cm layer in AG, which had the highest C:N ratio (12.89) in Autumn, while there was a decreasing trend in LF, which had the highest C:N ratio (15.91) in the 10–20 cm layer in Spring. There was no significant change in CT among soil depths and across three seasons. The soil C:N ratio in CT and LF was generally significantly higher than that in AG and CT, except in the 0–10 cm layer in Spring and Summer, while it was the reverse in Autumn (*p*<0.05).

**Fig 7:**
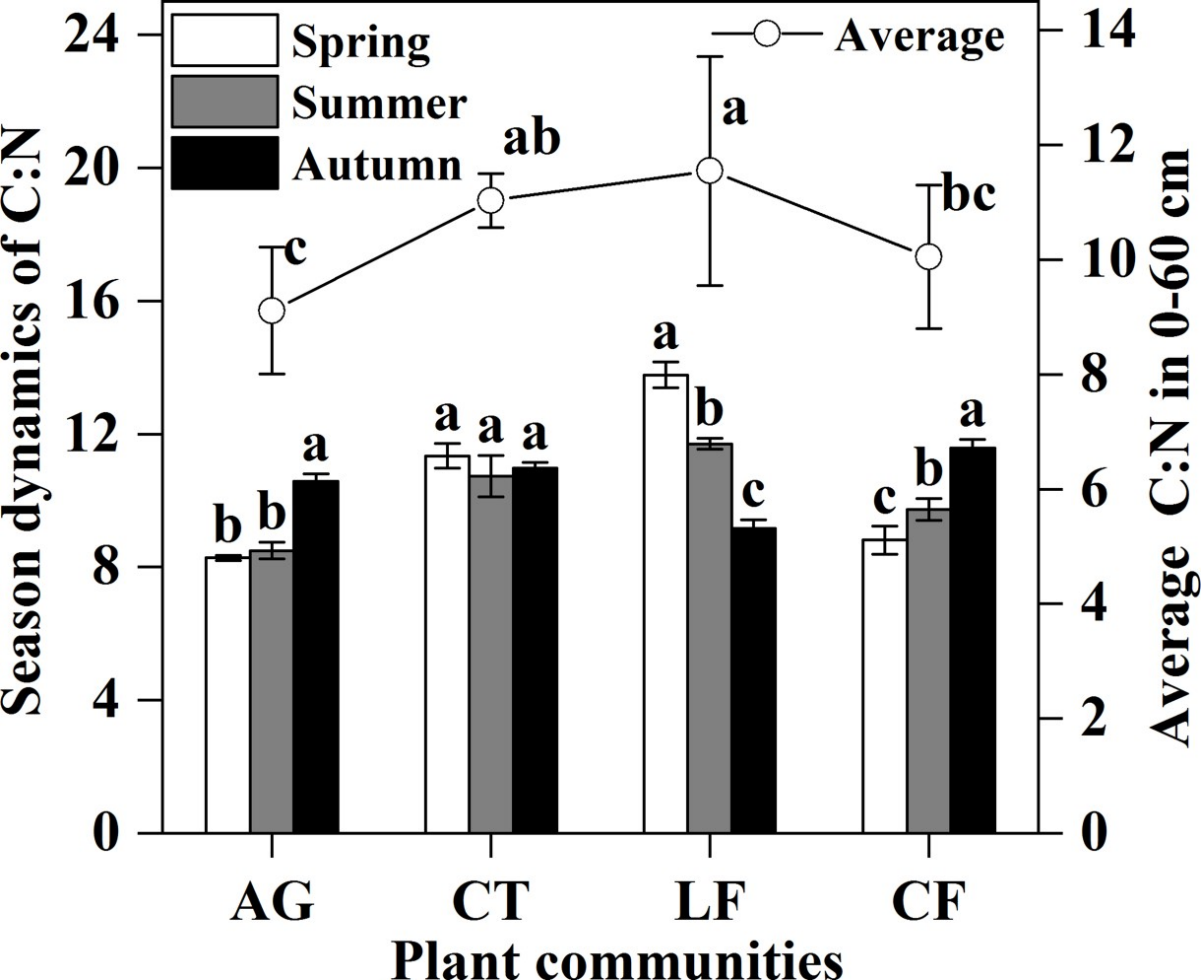
Seasonal dynamics of C:N ratio among plant communities and average C:N ratio. Bars indicate a significant difference (n = 4, *p*<0.05) between different seasons, and average concentration is between plant communities, as determined by ANOVA; AG: alfalfa grassland, CT: Chinese tamarisk, LF: locust forest and CF: cotton field.

The average C:N ratio (Fig 6*D*) in the 0–30 cm layer exhibited a different changing trend, while there was a steady trend in the 30–60 cm layer, with the lowest C:N ratio in AG. The seasonal dynamics (Fig 7) showed the same changing trend in AG and CF, with the highest C:N ratio in Autumn, while there was a different changing trend in LF with a significantly decreased C:N ratio from Spring to Autumn. The C:N ratio was not significantly different in CT with seasonal changes.

### Correlation among soil properties

**Fig 8:**
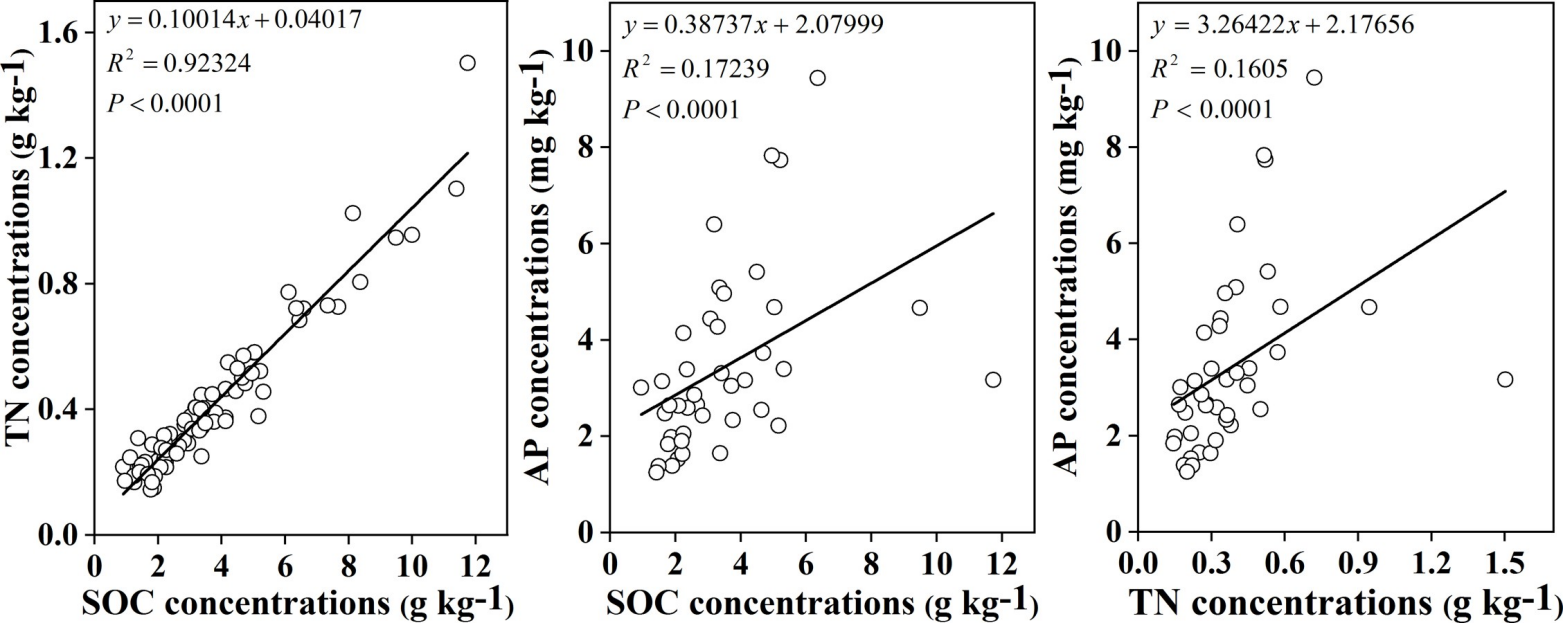
Correlation relationships between C, N and P among plant communities in the YRD.

Fig 8 showed that there was significantly positive relationship between SOC and TN; the same relationship was observed between SOC and AP and between soil TN and AP (Fig 8), but the positive relationship was not strong.

The correlation coefficients showed a significantly positive correlation among SOC, TN, C:N, AP and AK, which indicated that soil nutrients interacted with one another (Table 1). However, the pH was always negatively correlated with soil properties in the study area, it was significantly correlated with SOC (*p*<0.05) and with EC, C:N and AK (*p*<0.01). EC was positively correlated with AK (*p*<0.01).

**Table 1:** Correlation coefficients of soil properties (n = 24).

	pH	EC (us.cm^-1^)	SOC (g kg^-1^)	TN (g kg^-1^)	C: N	AP (mg kg^-1^)	AK (mg kg^-1^)
pH	1						
EC	-0.753**	1					
SOC	-0.476*	0.221	1				
TN	-0.360	0.142	0.980**	1			
C:N	-0.645**	0.202	0.381	0.217	1		
AP	-0.395	0.383	0.762**	0.723**	0.297	1	
AK	-0.707**	0.532**	0.788**	0.716**	0.436**	0.602**	1

Note:

**Correlation is significant at the 0.01 level (2-tailed)

* Correlation is significant at the 0.05 level (2-tailed).

Two-way ANOVA showed that soil depth had a significant effect on SOC and TN concentrations (*p*<0.001), as did the interaction among soil depth, plant communities and season (Table 2). The results indicated that soil vertical distribution was an important factor influencing SOC and TN.

**Table 2:** Results of two-way ANOVA for the effects of plant community, soil depth and seasonal dynamics on SOC and TN distribution (n = 216).

	df	SOC		TN	
		*F*	*P*	*F*	*P*
Plant community	3	1.696	0.214	0.512	0.681
Soil depth	5	23.119	<0.001	18.305	<0.001
Season	2	4.896	0.199	6.606	0.109
plant community × soil depth	15	4.771	<0.001	5.098	<0.001
plant community × season	6	1.255	0.307	0.987	0.452
soil depth × season	10	0.478	0.891	0.789	0.639
plant community × soil depth × season	30	4.276	<0.001	4.247	<0.001

## Discussion

The concentrations of soil C, N and P had differences among plant communities, soil depth and seasons in the scale of YRD. The higher SOC and TN concentrations occurred in forests (CT and LF), particularly in the topsoil layer of 0–10 cm. The higher concentrations of soil TN were observed in LF and CT with 1.50 g kg^-1^ and 1.10 g kg^-1^, respectively. Other TN concentrations were under 1.00 g kg^-1^, and the SOC and TN concentrations gradually decreased with the increase of soil depth. These results were in accordance with some studies that observed higher C and N concentrations in the topsoil [35] and indicated that higher concentrations of SOC and TN were related to the types of plant community, because the different plant communities had different amounts of plant residues above and below-ground returned to the soil [34, 36]. Some studies have also confirmed that the concentrations and vertical distribution pattern of soil C, N and P in wetland ecosystems were affected by the types of plant community [34]. The type of plant community, plant diversity and the presence of dominant species had differences in root distribution, the amount of litter returned to soil and plant cycling [21, 37]. The low SOC concentrations in alfalfa grassland (AG) and cotton field (CF) may be caused by crop harvests reducing the organic matter to the soil, because alfalfa was a main livestock feed source and cotton was a main cash-crop at the local scale. The alfalfa grassland also did not exhibit the advantage of nitrogen-fixing legumes, this result may have been affected by soil salination and the interactions between fluvial and marine processes in wetland ecosystem [21]. The seasonal variation of average SOC was limited mainly by temperature, the low temperature in winter greatly limited the activity of organisms [35]. Some studies have indicated that arable landscapes generally maintain low levels of soil C and N, mainly due to lower belowground productivity than that of perennial-dominated native prairie ecosystems [38, 39]. The higher AP in CT and CF was essentially in agreement with many studies that soil AP significantly increased after the conversion from natural wetlands to arable lands [40]. But it was worth noting that soil AP concentrations were generally low in this study site, the result was in accordance with others’ research results in the YRD (AP < 10 mg kg^-1^), which was in the level of grade four or below according to the data of the second national soil survey of China [41], and phosphorus in soil existed mainly in the form of medium steady state, the concentration of AP was low accounting for only 3.2%-5.9% of total phosphorus (TP) in soils [42]. Moreover, the percentage of adsorption was 70%-99%, which might restrict the growth of plants in the wetland system [42, 43].

The average soil C:N ratios in the study were generally lower than the mean values reported for Chinese soil in the 0–10 cm depth range (14.4) and as deep as 250 cm (11.9) [44], as well as in the *D*. *angustifolia* wetlands (12.97) and in *C*. *lasiocarpa* wetland soil (12.80) of the Sanjiang Plain for the 0–30 cm depth [28, 34]. The result indicated that the return of organic matter to soil in the YRD was relatively low, because the plants are the major source of soil C and N in terrestrial ecosystems, and some studies showed that the soil in the YRD scale was in a “N limitation” state [45]. The average C:N ratio values of three seasons were higher in LF(11.55) and CT(11.03) than that in CF(10.05) and AG(9.11) in 0–60 cm layer, which demonstrated that there were relative more residues returned to the soil in natural forests than in reclaimed land, this result supports Cleveland and Liptzin’s hypothesis that soil C:N ratio may be comparatively consistent at large spatial scales [46] while the C:N ratio existed a certain degree of heterogeneity at the local scale [28]. The C:N ratio varied greatly with soil depth in this study, because soil profile depth, variations in soil properties and the activities of microorganisms can have a substantial effect on the C:N ratio [47, 48] at smaller scales or in the types of plant communities due to land uses [28, 45].

The correlations indicated that soil nutrients interacted with one another, but pH always played a negative role in soil nutrients (Table 1). There was a strong significant positive correlation between soil C and N (Fig 8), it demonstrated that C was generally closely related to N in soil [28]. The correlation was not strong between C and P or between N and P, because soil P in natural wetlands mainly depends on the mineral weathering and soil development of the parent material [45], which led to the lower P levels in the YRD. Some results identified that the characteristics of saline-alkali soil in the YRD were low organic matter content, lack of nitrogen, poor phosphorus and rich potassium [49]. Therefore, combining our research data and the characteristics of the YRD, we suggest that protecting natural vegetation and developing optimal plant communities are critical tasks for improving soil quality and mitigate land degradation in the YRD. The study results were from a field investigation for a certain temporal and spatial scale, and the mechanism study of how plant communities and coeffects of environmental factors influence soil C, N, and P and their stoichiometry needs to be confirmed through further research on large temporal and spatial scales in the wetland ecosystems.

## Conclusions

Results of this study indicated that soil C, N and P have different profile distribution, reflecting the differences in nutrient input by plant communities in the YRD. The higher concentrations of SOC and TN in CT and LF indicated that there was a potential to increase SOC and TN in forests. The reclaimed land had low SOC and TN due to soil carbon and nitrogen loss following the conversion from natural vegetation to farmland community at a certain temporal scale. The lower C:N ratios in reclaimed land demonstrated a negative effect on SOC and TN accumulation and low residues return. The soil nutrients interacted with one another and had a certain degree of heterogeneity affected by plant communities at the local scale. Therefore, protecting natural vegetation and developing optimal plant communities are critical tasks to improve the soil quality and to mitigate land degradation in the YRD.

## Supporting information

S1 File Data for the cited Figs 2–8 in the manuscript. (XLS)

S2 File Data for the cited Tables 1 and 2 in the manuscript. (XLS)
